# On the static structural design of climbing robots: part 1

**DOI:** 10.1186/s40638-015-0030-y

**Published:** 2015-12-01

**Authors:** Ausama Hadi Ahmed, Carlo Menon

**Affiliations:** MENRVA Research Group, School of Engineering Science, Simon Fraser University, Burnaby, BC V5A 1S6 Canada

**Keywords:** Climbing robot, Geometry design, FEM

## Abstract

This manuscript is the first of two parts of a work investigating optimal configurations of legged climbing robots while loitering on vertical surfaces. In this part 1, a mathematical model of a climbing robot based on the finite element method (FEM), specifically the stiffness method, is generated. A number of parameters, namely the height of the robot, the length of its body and the position of its legs, are investigated to assess their effect on the adhesion requirements needed for the robot to stay attached to a wall. Predictions of the developed mathematical model are validated using FEM commercial software. The body and the legs are assumed to be perpendicular to each other in this part 1. The effect of their inclination is investigated in the subsequent part 2 of our work. In part 2, the model is also used to predict postures that ants have while standing on vertical surfaces. The model is validated by comparing the predicted results to images of loitering ants. The parameters investigated provide guidelines to design legged climbing robots.

## Background

A variety of legged robots having different size and design have been proposed in the literature to climb vertical surfaces. Such robots have different locomotion systems including tracks [1–3], wheels [4, 5], and legs [6, 7]. Legged robots, which are of primary interest for this work, use a variety of mechanisms to adhere to climbing surfaces, including magnets [8], dry adhesion, in the form of single layer [9, 10] or hierarchical [11], spines/claws [12–15], and negative pressure [16].

The design of some climbing robots was inspired by nature’s living organisms including geckos [7], spiders [6], cockroaches [12] or a combination of different species such as geckos and cockroaches [17]. The arrangement and inclination of the legs are not the same among the different designs of the robots. In fact, some of the robots, including Spinybot II [15], had their legs inclined forward; others, including Abigaille III [11] and ROBUG II [8], have some of their legs inclined forward and some backward; some others, including the RiSE and DIGbot [12, 18], have their legs on the sides of their bodies, and others, including Abigaille II [6], have legs symmetrically distributed around their bodies.

In this work, the size and the configuration of legged climbing robots are analyzed by investigating the effect that different design parameters have on the maximum attachment force required by the robot to stay attached to a vertical surface. This work investigates the design of six-legged robots. They are analyzed in a 2-D space by taking into account the geometrical symmetries hexapods generally have. The finite element method (FEM) is used to analyze the force distribution on the tips of the robotic legs; FEM is selected as it provides more accurate results than approximated quasi-static methods generally used to investigate this problem [1, 6, 9–11, 19–21]. In this work, dimensionless parameters are selected as the beams used in the structures investigated in this work are scalable as long as the second moment of inertia is fixed.

The amount of force available to the robot to adhere to a vertical surface is critical regardless of the type of attachment mechanism (e.g., dry adhesion, suction, magnets) used to climb. A successful climbing robot should, in fact, always have enough force to be able to adhere to the wall. In this work, the optimal structure of the robot is considered to be the one that requires the least attachment force to adhere to a vertical wall. This optimal structure would maximize the safety factor to avoid detachment or equivalently minimize the adhesion strength required by the adhesive pads or grasping mechanisms used by the robot to adhere to a vertical surface. The formulation developed in this work is, therefore, general and is suitable to model legged robots relying on a large variety of different sources of adhesion.

This manuscript is organized as follows: “Kinematics” section presents the model and the kinematics of the examined multi-legged structure; “Structural analysis” section describes the proposed method to analyze the force distribution of the robot; “Investigated parameters” section presents results obtained by changing the different geometrical parameters of the considered structure on the force distribution on the tips of the robot’s legs. Conclusions and recommendations for the design of climbing legged robots are drawn at the end of the manuscript.

## Kinematics

**Fig. 1:**
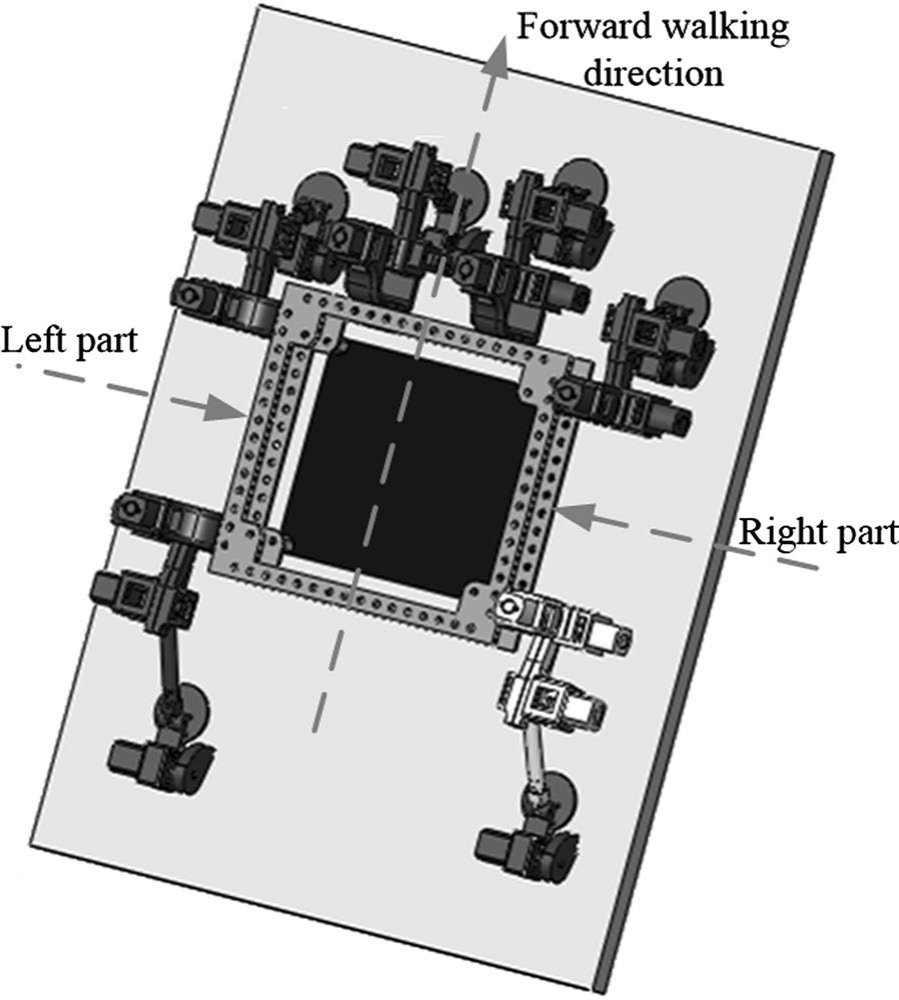
Abigaille III [3] walking upward of a surface

Hexapod robots such as Digbot [12], Abigaille II [6] and Abigaille III [3] generally have an axis of symmetry parallel to the forward walking direction, shown in Fig. 1. Such robots can be simplified and studied in 2-dimensions, because the left and the right parts of the robots are symmetric.

**Fig. 2:**
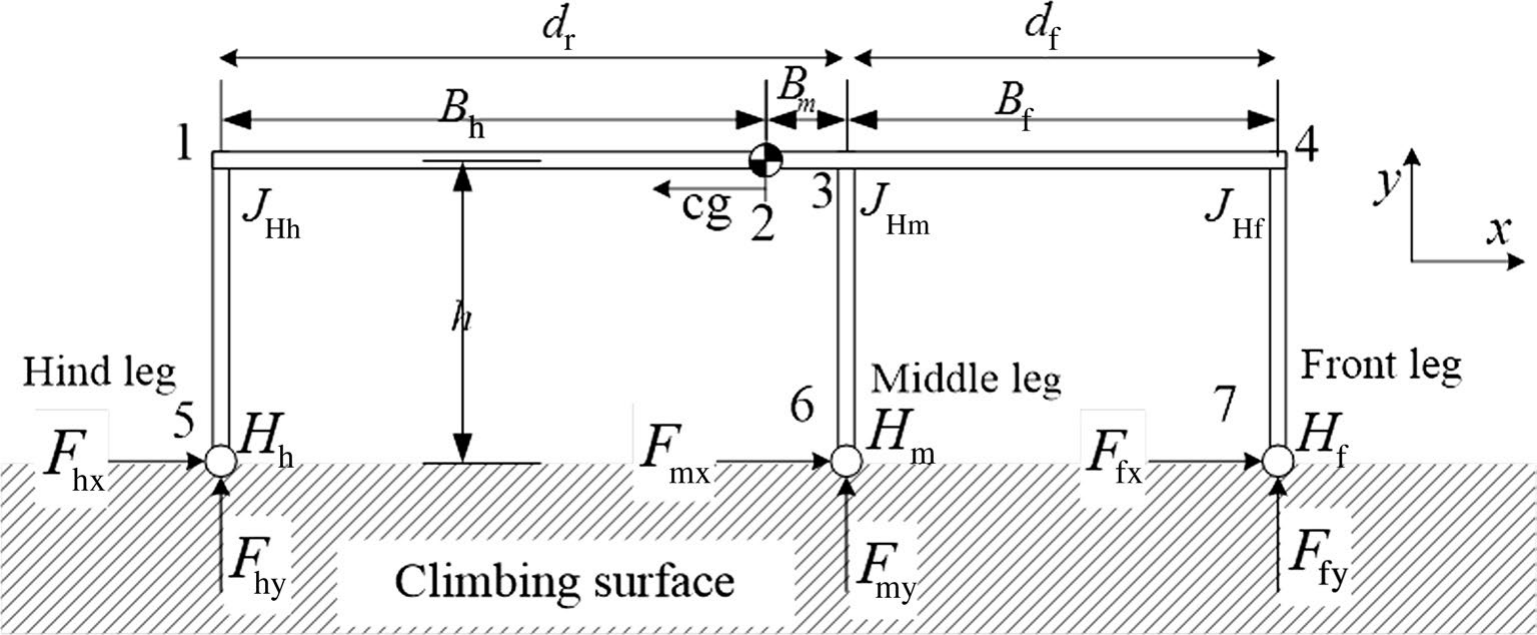
The 2D simplified model of Abigaille III [11]

In this work, the robot is considered to be loitering, as it is attached to the vertical surface. In this configuration, the motors of a robot would exert a constant torque on their legs to keep them in place and avoid detachment. From a quasi-static analysis perspective, each leg can, therefore, be considered as a part of a rigid structure. To simplify the analysis and draw conclusions that could be generalized to most six-legged robots, each robotic leg was arbitrarily simplified to be a straight equivalent beam, with stiffness approximately equal to that of the robotic leg. To account for the different possible values of stiffness that different robots or different leg’s configurations could have, we varied the cross-sectional area of the equivalent beam. A similar consideration was done for the body of the robot, which was also modeled with a straight beam and whose stiffness was changed by changing its cross-sectional area. By considering the legs and body weightless and assuming the mass of the robot to be concentrated at its centre of mass (CoM), which is consistent with the existing literature [6, 9–11, 21–25], the variation of the cross-sectional area did not affect the weight of the robot and a comparative analysis was, therefore, possible. It should be noted that the effect of taking the weight of the legs into account without changing the overall weight of the robot would only slightly affect the shear and normal force distribution in the feet. Specifically, the shear forces would be more evenly distributed among the legs. The normal forces on the feet would instead slightly decrease, given the center of mass of the robot would be closer to the surface. In this work, the weight of the robot is assumed to be equal to one unit in all of the performed calculations in order to conveniently represent the forces on the tips of the feet as a percentage of the applied load. This normalization is used to generalize the results obtained in this work to a large variety of robots having different values of weight and dimensions. Figure 2 shows the simplified equivalent model that was considered. It should be noted that the legs of the robot were assumed to not transfer moment to the vertical surface, as commonly done in the literature [6, 9–11, 21, 24–27].

It should be noted that while this article specifically addresses robots in a static configuration, results of this work could be generalized to a certain extent to dynamic systems, as inertial forces resulting from accelerations of the robot would simply add to the weight of the robot, without affecting the optimal geometries investigated in this work. Variation of posture during walking is not addressed in this work as it resides outside the scope of this study.

## Structural analysis

The robotic structure presented in “Kinematics” section is analyzed using the stiffness method [28], which uses the beams’ stiffness relations to compute the forces and the displacements of the structure. The overall relationship between the forces applied to the structure (axial loads, shear loads and bending moments) and the resulted displacements is given by

1 $$\varvec{F} = \varvec{KD}$$

where *K* is the structural stiffness matrix, $F$ is a vector representing both the known forces applied to the structure and the unknown reaction forces of the nodes and $D$ is a vector comprising the known and the unknown displacements of the nodes. Damping is not included as a static analysis is considered in this work.

The structure of the robot is divided into six separate beams, see Fig. 2. Specifically, each of the three legs, the connection between the hind leg and the center of mass, the connection between the center of mass and the middle leg and the connection between the middle leg and the front leg are all considered to be separate beams. The case when the middle leg is located between the hind leg and the center of mass is also formed using six beams: specifically, the three legs, the connection between the hind leg and the middle leg, the connection between the middle leg and the center of mass and the connection between the center of mass and the front leg.

The known displacements are those of the constrained nodes, namely those of the hinges ($H_{\text{h}} , H_{\text{m}} , H_{\text{f}}$) in *x* and *y* axes (see Fig. 2), are equal to zero. The unknown degrees of freedom are the distance the unconstrained nodes moved after applying the known forces on the structure; from Fig. 2, the unknown degrees of freedom are the linear movement of nodes 1, 2, 3 and 4, and the rotation movement of all of the nodes, namely nodes 1–7. The known forces are the weight of the robot at the center of mass, and the linear force components of all of the unconstrained nodes, namely nodes 1–4 along with the moment on all of the nodes, namely nodes 1–7, are equal to zero. The unknown forces are the reaction forces at the hinges, namely $F_{\text{hx}} , F_{\text{hy}} , F_{\text{mx}} , F_{\text{my}} , F_{\text{fx}}$ and $F_{\text{fy}}$, which are shown in Fig. 2. Equation (1) can, therefore, be written as:

2 $$\left[ {\begin{array}{*{20}c} {\varvec{F}_{{\mathbf{k}}} } \\ {\varvec{F}_{{\mathbf{u}}} } \\ \end{array} } \right] = \left[ {\begin{array}{*{20}c} {\varvec{K}_{11} } & {\varvec{K}_{12} } \\ {\varvec{K}_{21} } & {\varvec{K}_{22} } \\ \end{array} } \right]\left[ {\begin{array}{*{20}c} {\varvec{D}_{{\mathbf{u}}} } \\ {\varvec{D}_{{\mathbf{k}}} } \\ \end{array} } \right]$$

where $\varvec{F}_{{\mathbf{k}}}$ is the vector of the known forces, $\varvec{F}_{{\mathbf{u}}}$ is the vector of the unknown forces, $\varvec{D}_{{\mathbf{u}}}$ is the vector of the unknown displacements and $\varvec{D}_{{\mathbf{k}}}$ is the vector of the constrained displacements.

From Eq. (2), the unknown displacements $\varvec{D}_{\text{u}}$ can be calculated as follows:

3 $$\varvec{D}_{{\mathbf{u}}} = \varvec{K}_{11}^{ - 1} \cdot \varvec{F}_{{\mathbf{k}}} - \varvec{K}_{11}^{ - 1} \cdot \varvec{K}_{12} \cdot \varvec{D}_{{\mathbf{k}}}$$

The unknown forces that are the reaction forces between the tips of the legs and the climbing surface are calculated using

4 $$\varvec{F}_{{\mathbf{u}}} = \varvec{K}_{21} \cdot \varvec{D}_{{\mathbf{u}}} + \varvec{K}_{22} \cdot \varvec{D}_{{\mathbf{k}}}$$

Substituting Eq. (3) into Eq. (4) yields:

5 $$\varvec{F}_{{\mathbf{u}}} = \varvec{K}_{21} \cdot \left[ {\varvec{K}_{11}^{ - 1} \cdot \varvec{F}_{{\mathbf{k}}} - \varvec{K}_{12} \cdot \varvec{D}_{{\mathbf{k}}} } \right] + \varvec{K}_{22} \cdot \varvec{D}_{{\mathbf{k}}}$$

The known distances $\varvec{D}_{\text{k}}$ are the displacements of the constrained nodes which are equal to zero; as such, the above equation can be rewritten as:

6 $$\varvec{F}_{{\mathbf{u}}} = \varvec{K}_{21} \cdot \varvec{K}_{11}^{ - 1} \cdot \varvec{F}_{{\mathbf{k}}}$$

Equation (6) is a closed form equation to calculate the reaction forces. Such an equation is implemented on a code developed in MATLAB environment. It should be noted that the force distribution depends on the stiffness of each beam relative to the other beams and not to the absolute stiffness value of each beam (see “Appendix”). Therefore, the results obtained in this work can be generalized to robots having any material and stiffness.

Commercially available finite element method (FEM) software, i.e., ANSYS (V14.0), is used to verify the correct implementation of the stiffness method. Specifically, the beam element BEAM188 based on Timoshenko beam theory is used to analyze 2D structures [29]. The MATLAB code is tested against the ANSYS software by comparing randomly selected cases solved using MATLAB with the same cases solved using ANSYS.

## Investigated parameters

The FEM presented in “Structural analysis” is used in this section to minimize the normal adhesion required by the robot to stay attached to a vertical surface, i.e., $F_{\text{hy}} , F_{\text{my}}$ and $F_{\text{fy}}$ in Fig. 2. The normal force is investigated by examining different parameters of the structure with the assumption that the legs are always perpendicular and the body is parallel to the climbing surface. The investigated parameters are: (1) the position of the middle leg; (2) the body height to body length aspect ratio; (3) the cross-sectional area of the beams forming the body and the legs. These three parameters are investigated in the following sections in pairs. Specifically, first the parameters 1 and 2 are investigated while parameter 3 is fixed; subsequently, the parameters 1 and 3 are investigated while parameter 2 is fixed. The combined effect of the three parameters is generalized in a later section.

### Effect of height to length ratio and middle leg’s position

**Fig. 3:**
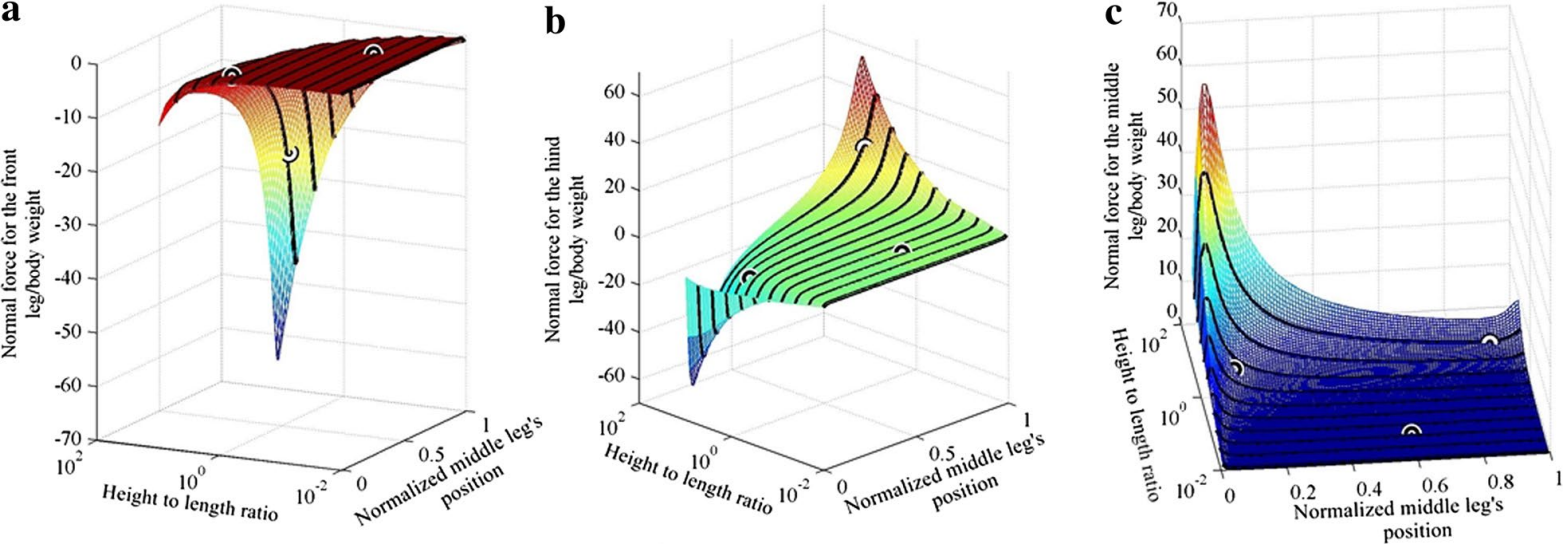
The required normal force at different height to length aspect ratio and different middle leg’s positions for **a** front leg, **b** middle leg and **c** hind leg. *Circles* represent simulations performed using ANSYS

This section describes the effect of changing the body height to body length aspect ratio and the effect of the position of the middle leg on the adhesion force requirement on the tips of the legs. For the robot shown in Fig. 2, $d_{\text{f}}$ is the distance between the middle and the front legs, $d_{\text{r}}$ is the distance between the middle and the hind legs and $h$ is the height of the robot. The length of the body, $d_{\text{f}} + d_{\text{r}}$, is arbitrarily chosen to be 200, while the radius of the beams is assumed to be the same and equals to two, and the height is in the 2.1–2000 range, which corresponds to a range of height to body length ratio of 0.0105–10. The obtained results are applicable to both bigger and smaller structures as long as the ratio of the height to the length is within the range and the radius is kept fixed. Distribution of the calculated normal force, representing the adhesion force requirement, for the change in the height to length aspect ratio and the position of the middle leg is shown in Fig. 3. Three different configurations are compared with ANSYS and plotted over the curve (see circles in Fig. 3) obtained using MATLAB. The average error between simulations performed in MATLAB and ANSYS is 0.61 %.

The *x* axis in Fig. 3 represents the position of the middle leg, where 0 means that the middle leg is positioned at the back of the robot. In this configuration, the middle leg has the same position as the hind leg. The value of the *x* axis increases as the middle leg gets closer to the front leg and the value equals 1 when the middle and front legs have the same position. The *y* axis represents the height to length aspect ratio and the *z* axis represents the normal force per body weight. From Fig. 3, increasing the body height to body length ratio, on the *x* axis, requires higher force to keep the robot attached to the vertical surface because, a robot with higher height and fixed weight causes an increase in the torque applied to the robot’s structure due to gravity which needs higher forces on the tips of the legs to keep the robot in equilibrium than that required by a robot with lower height.

The normal force in Fig. 3 has two peaks located at middle leg positions of 0.07 and 0.93; the first peak is located at the hind leg with a maximum of 64.16 and the second is located at the middle leg with a maximum of 56.66. The peaks can be explained by analyzing the shear and normal forces distributions for a specific robotic structure with fixed height to length aspect ratio. The shear force distribution due to changing the middle leg’s position is explained first and the normal force distribution resulting from changing the middle leg’s position is explained next.

#### Shear force distribution due to middle leg’s position

**Fig. 4:**
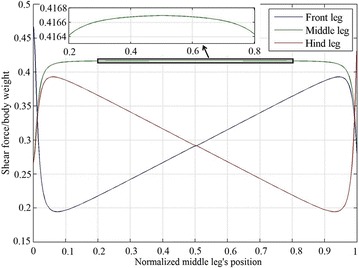
Shear force distributions on the tips of the three legs for different positions of the middle leg

A structure with a height to body length aspect ratio of 1:2 is arbitrarily selected to explain the behavior of the normal force distribution due to changing the middle leg’s position. The shear force distribution on the legs of a robot with body length of 200, and height of 100 is shown in Fig. 4. The behavior of the force distribution for a three-legged robot is similar for different height to length ratios. The shear force distribution for the middle leg always has a peak at middle leg’s position of 0.5, while the front leg has a maximum at middle leg’s position of 0, and the hind leg has a maximum at middle leg’s position of 1. The normal force, in Fig. 3, for the middle leg has a minimum and a maximum at middle leg’s position value close to 0 and 1, respectively, the front leg has one peak close to middle leg’s position of 1 and the hind leg has one negative peak at a middle leg position of 0.

**Fig. 5:**
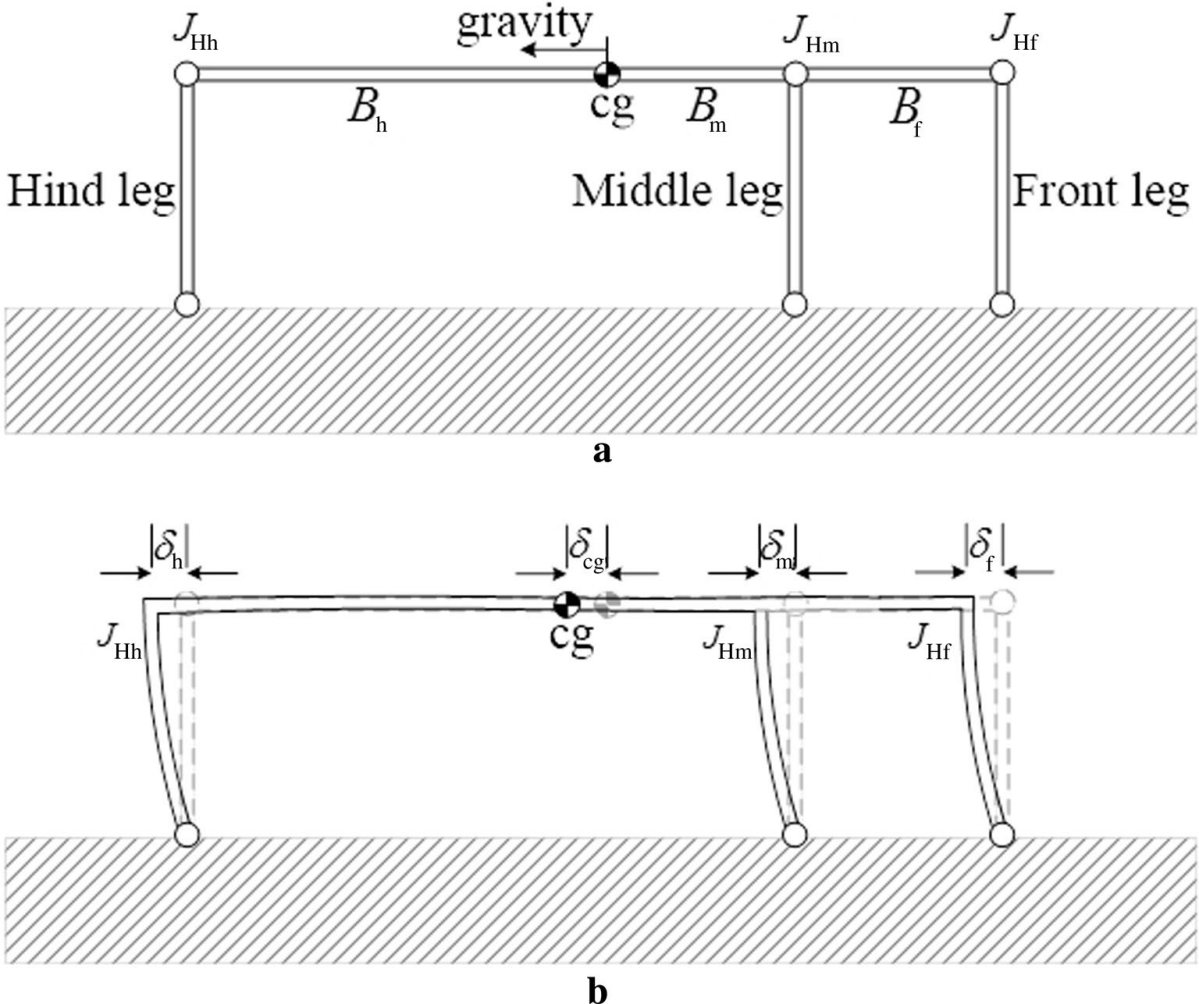
The deflection in the robot’s structure due to its weight when loitering on a vertical surface, **a** the un-deflected structure, and **b** the deflected structure

A rationale to understand the behavior shown in Fig. 4 is hereafter presented. Let us consider a robot on a vertical surface (see Fig. 5a). Due to the effect of its weight, the legs deflect backward and act as springs with equal spring constants. Therefore, the $cg$, the hip joints of the front leg $(J_{\text{Hf}} )$, the middle leg $(J_{\text{Hm}} )$ and front leg $(J_{Hh} )$ are displaced backward by a distance $\delta_{\text{cg}} ,\delta_{\text{f}} , \delta_{\text{m}}$ and $\delta_{\text{h}}$, respectively (see Fig. 5b). The induced shear forces on the tips of the legs are directly proportional to the displacements $\delta_{\text{h}} , \;\delta_{\text{m}}$ and $\delta_{\text{f}}$, because the legs are assumed to be identical to each other.

Figure 5, which is obtained through an ANSYS simulation, shows the deflections in the structure. In Fig. 5, $B_{\text{m}}$ is the beam connecting $J_{\text{Hm}}$ to ${\text{cg}}$. $B_{\text{f}}$ and $B_{\text{h}}$ are instead the beams connecting $J_{\text{Hf}}$ to $J_{\text{Hm}}$ and $J_{\text{Hh}}$ to $cg$, respectively, when the middle leg is located between ${\text{cg}}$ and $J_{\text{Hf}}$. These two parameters, that is $B_{\text{f}}$ and $B_{\text{h}}$, are the beams connecting $J_{\text{Hf}}$ to $cg$ and $J_{\text{Hh}}$ to $J_{\text{Hm}}$, respectively, when the middle leg is located between $cg$ and $J_{\text{Hh}}$.

When the middle leg is located between the center of mass and the front leg, the body’s deflection creates a compression in $$B_{\text{m}}$$ and $$B_{\text{f}}$$ and an expansion in $$B_{\text{h}}$$, thus causing the distances $$\delta_{\text{f}} ,$$$$\delta_{\text{m}}$$ and $$\delta_{\text{h}}$$ to be less than $$\delta_{cg}$$. The distance $$\delta_{\text{h}}$$ is equal to the compression in $$B_{\text{h}}$$ subtracted from $$\delta_{cg}$$; also, $$\delta_{\text{m}}$$ is equal to the elongation in $$B_{\text{m}}$$ subtracted from $$\delta_{cg}$$, and $$\delta_{\text{f}}$$ is equal to the compression in $$B_{\text{f}}$$ subtracted from $$\delta_{\text{m}}$$.

The maximum distance that $J_{\text{Hm}}$ travels is when it is located at the center of mass $cg,$ which corresponds to the maximum force it experiences. The expansion in $B_{\text{f}}$ and the compression in $B_{\text{h}}$ cause $\delta_{\text{f}}$ and $\delta_{\text{h}}$ to be less than $\delta_{\text{m}}$; these expansion and compression generate less shear force in the hind and the front legs than that in the middle one (see Fig. 4 when the middle leg’s position is at 0.5). The front and middle legs have the same shear force when the middle leg’s position is at 1, because $\delta_{\text{m}}$ and $\delta_{\text{f}}$ are equal. The amount of expansion in $B_{\text{f}}$ increases with the length, causing $\delta_{\text{f}}$ to be smaller than $\delta_{\text{m}}$ and thus generating less shear force in the front leg than that in the middle one (see Fig. 4 for a middle leg’s position ranging between 0.5 and 1).

The case when the middle leg is located between the center of mass and the hind leg could be analyzed as done previously. The main difference is that the beam $B_{\text{m}}$ undergoes compression instead of expansion.

#### Normal force distribution due to middle leg’s position

**Fig. 6:**
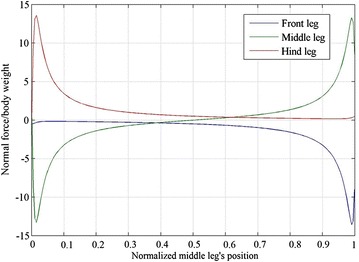
Normal force distribution on the tips of the legs for different middle leg’s positions. The maximum adhesion force follows the profile of the *green line* in the 0–0.38 range and the *blue line* in the 0.38–1 range

The normal force distribution at different middle leg’s positions is shown in Fig. 6. The distribution of the force on the tips of the legs can be explained by dividing the robot into two sub-structures at $J_{\text{Hm}}$. The first sub-structure, when the middle leg’s position is located between 0.5 and 1, is composed of the middle leg, $B_{\text{f}}$ and the front leg while the second sub-structure is composed of $B_{\text{m}} ,\; B_{\text{h}}$ and the hind leg. The first sub-structure, when the middle leg is positioned between 0 and 0.5, is composed of the middle leg, $B_{\text{h}}$ and the hind leg and the second sub-structure is composed of $B_{\text{m}} , \;B_{\text{f}}$ and the front leg.

**Fig. 7:**
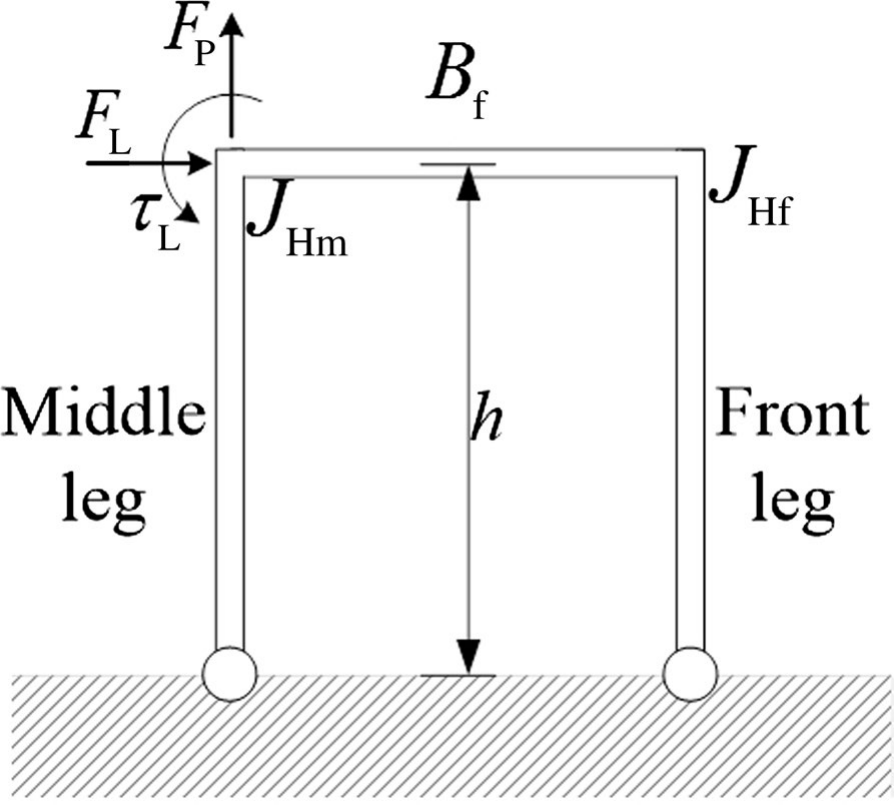
Structure of a 2-legged robot

The second sub-structure, formed by the hind leg, $B_{\text{h}}$ and $B_{\text{m}}$, when the middle leg’s position between 0.5 and 1, applies a wrench to the first sub-structure, formed by the middle and the front legs with $B_{\text{f}}$; the wrench is composed of $F_{\text{L}}$ which has the same direction as the gravitational force towards the negative *x* axis, a shear $F_{\text{P}}$ towards the positive *y* axis, and a torque $\tau$ applied at $J_{\text{Hm}}$. The effect of the wrench can be analyzed by considering the structure that contains the middle leg, the front leg and $B_{\text{f}}$ in Fig. 7. The effect of the individual components of the wrench is explained as follows:

First, the effect of $\tau_{\text{L}}$: applying a torque $\tau_{\text{L}}$ at $J_{\text{Hm}}$ causes a tension force to be generated in the front leg and a compression force in the middle leg. A decrease in the length of $B_{\text{f}}$, due to a change in the middle leg’s position towards the front, causes an increase in the tension and the compression magnitude in the front and middle legs, and vice versa.

Second, the effect of $F_{\text{L}}$: applying force $F_{\text{L}}$ at $J_{\text{Hm}}$ causes a tension force to be generated in the middle leg and a compression force in the front leg. A decrease in the length of $B_{\text{f}}$, due to a change in the middle leg’s position, causes an increase in the amount of the tension and compression, and vice versa. Third, the effect of $F_{\text{P}}$: applying force $F_{\text{P}}$ in the positive direction of the *y* axis at $J_{\text{Hm}}$ causes a tension force in only the middle leg, i.e., a decrease in the normal force of the middle leg.

Each of the first two wrench components, namely $\tau_{\text{L}}$ and $F_{\text{L}}$, causes opposite forces along the middle and the front legs, thus causing the big difference in the normal forces of the middle and front legs, which are colored green and blue in Fig. 6. The difference in the forces is a result of the opposite forces generated in the middle and front legs by $\tau_{\text{L}}$ and $F_{\text{L}}$.

**Fig. 8:**
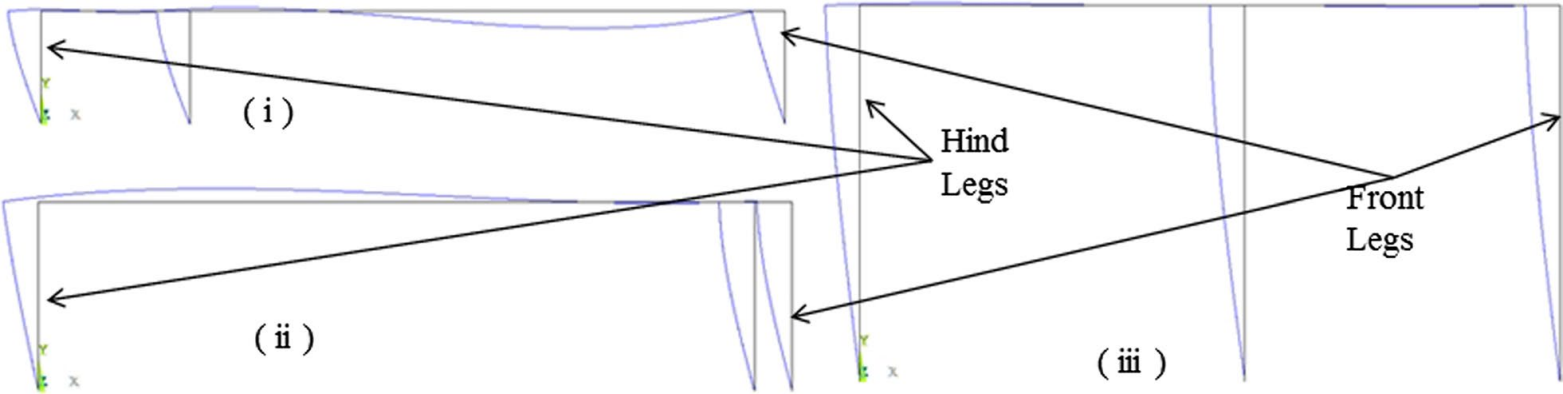
The deflection of the structure of a robot for different heights and middle leg’s positions. The body has body length of 200, elasticity of $1.12 \times 10^{9}$ and a unit weight. **i** The height is 30 and $\it d_{\text{r}}$ is equal to 40. Note that the deflection is magnified by $1.95 \times 10^{5}$ times. **ii** The height is 50 and $\it d_{\text{r}}$ is equal to 190. Note that the deflection is magnified by 626.7 times. **iii** The height is 100 and $\it d_{\text{r}}$ is equal to 110. The deflection is magnified by 8928.4 times

The case when the middle leg is positioned between 0 and 0.5 can be analyzed similarly. The second sub-structure, formed by $B_{\text{m}} , B_{\text{f}}$ and the front leg, applies a wrench to the first sub-structure, formed by the middle leg, $B_{\text{h}}$ and the hind leg, at $J_{\text{Hm}}$ while the normal force at the tips is a result of the wrench applied. The deflected shapes for different configurations of the robot are shown in Fig. 8.

#### Optimal middle leg position and height to length ratio

**Fig. 9:**
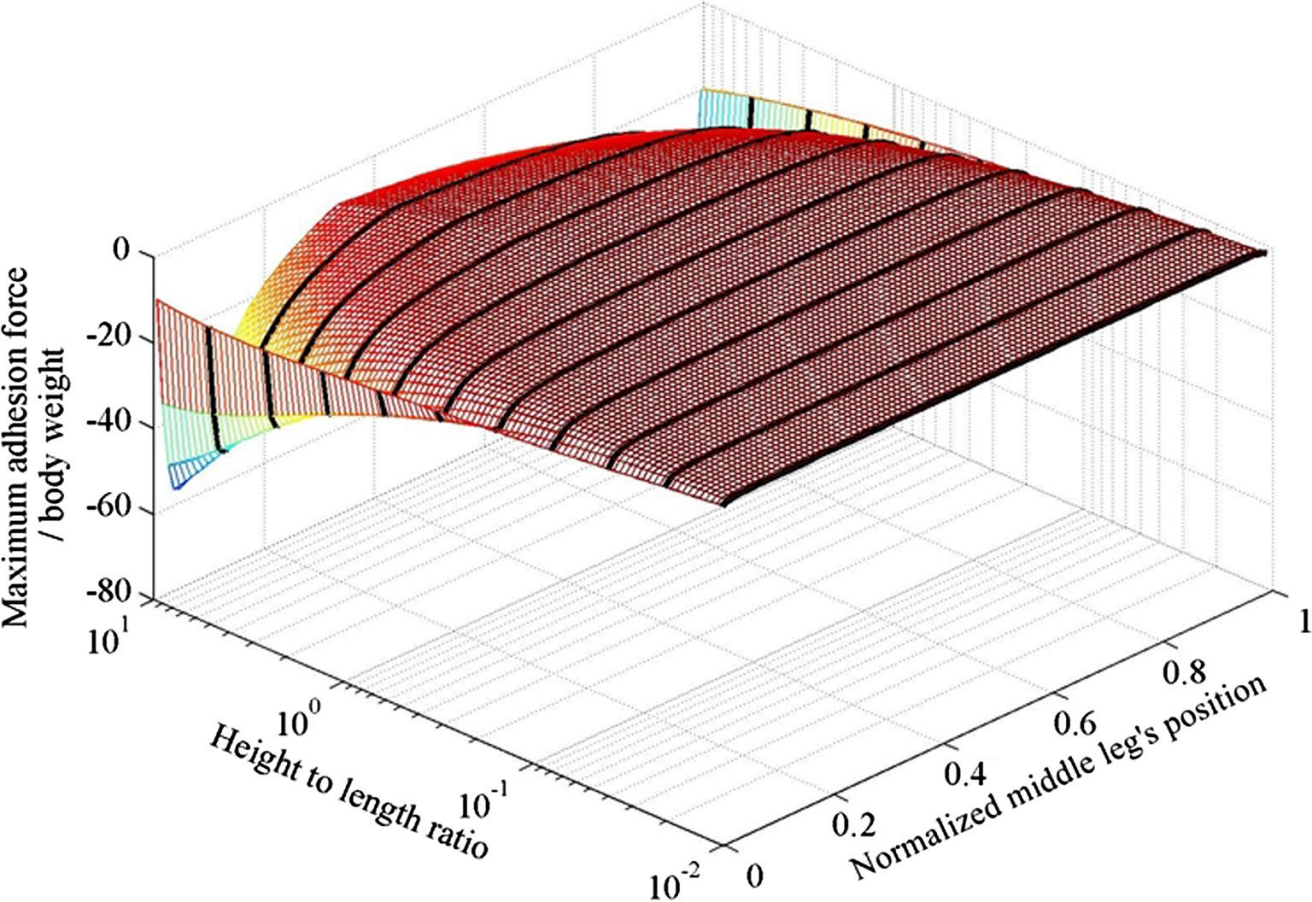
The maximum adhesion force for different height to length ratios and different middle leg’s positions

The design that requires the least adhesion force for a robot with parallel body and perpendicular to the climbing surface legs can be found in Fig. 3. The maximum adhesion needed by any of the legs at different heights and different middle leg’s positions is shown in Fig. 9. An optimization is performed to identify the optimal height and middle leg’s position of the robot; the optimal structure found has an optimal height of 2.1 and an optimal middle leg’s position at 0.335.

The optimizer is configured to search for the optimal middle leg’s position within the range of 0–0.95 to prevent the optimizer from converging to the global optimum at 1. In fact, the global optimum is not considered to be the most desirable value as a small variation from the minimum causes a dramatic increase in the adhesion requirement. In fact, in Fig. 6, a small variation of the leg from its optimal position causes the maximum adhesion requirement to increase dramatically. For example, a 0.01 variation in position causes a more than 250-fold increase in the required adhesion.

It should be noted that the 2.1 is the smallest height that is considered in this study. In fact, the radius of the structure is assumed to be two and a minimum gap between the robot and the surface is assumed to be 0.1. As expected, it can be concluded that the height should be as low as possible.

### Effect of cross-sectional area and middle leg position on force distribution

In this section, the effect of the stiffness of the legs is considered. Specifically, we investigate whether a robot should have stiff or compliant legs to minimize the adhesion force required to adhere to a vertical surface. To change the stiffness of the legs, their cross-sectional area was varied.

At first, a structure with body length of 200 and a height of 100 is arbitrarily chosen to explore the effect of changing the cross-sectional area on the normal force distribution of a robot. Results drawn from this specific geometry are generalized in a subsequent section.

The cross-sectional area and the area moment of inertia are varied, while the weight of the robot is considered to be fixed at one and applied at the center of mass. The area moment of inertia is calculated to be equivalent to that of a circle; the radius is calculated from the cross-sectional area, and the area moment of inertia is then calculated accordingly. The normal force distribution is calculated for different cross-sectional area values for the legs, between $0.0001\;{\text{and}}\;3.16 \times 10^{4}$, while keeping the cross-sectional area of the body, formed by the horizontal beams in Fig. 1, fixed at one. This analysis, therefore, explores if the legs should be more or less stiff than the body to minimize the maximum required adhesion.

**Fig. 10:**
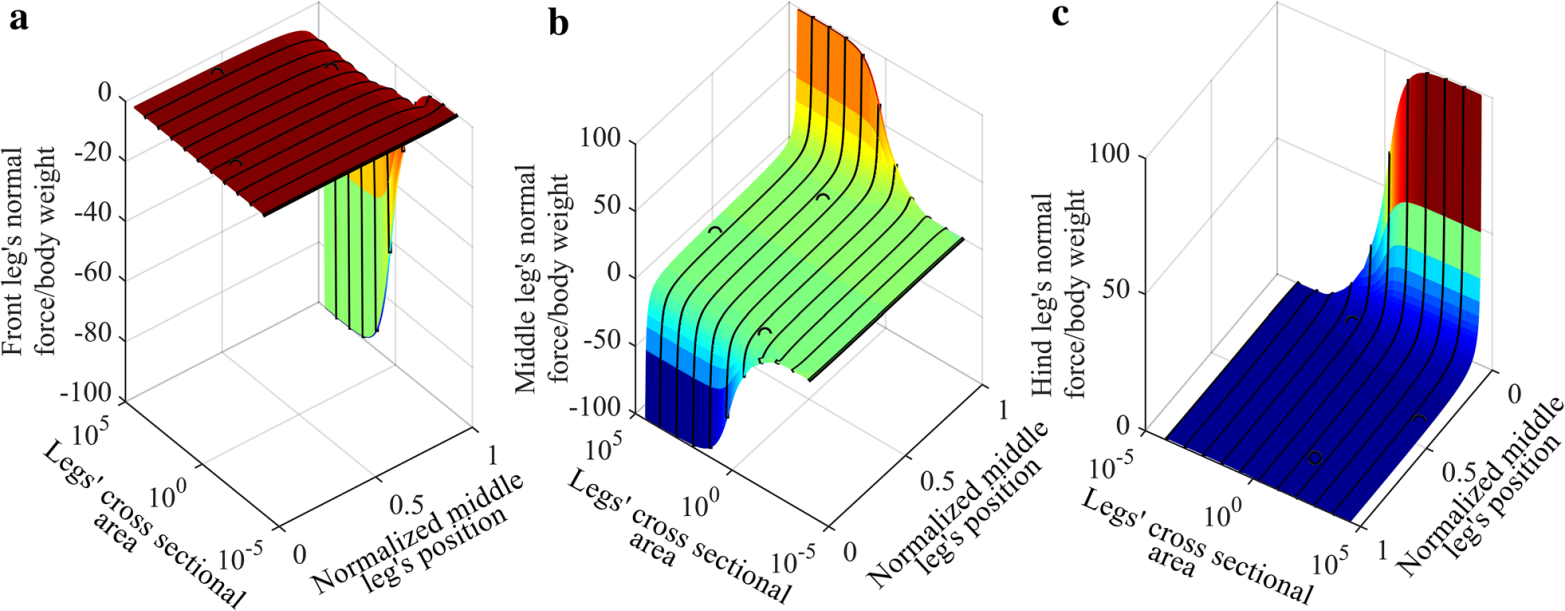
Normal forces required by the feet of the robot for different legs’ cross-sectional areas and different middle leg’s positions with the body’s cross-sectional area fixed at 1. *Circles* represent simulations performed using ANSYS

The normal force required for each leg to stick on the wall for different legs’ cross-sectional areas and middle leg’s positions is shown in Fig. 10. Three different configurations are compared with ANSYS and plotted over the curve obtained in Fig. 10; the ANSYS test points have a negligible error (an average absolute error of approximately 2 %) compared to our predictions.

**Fig. 11:**
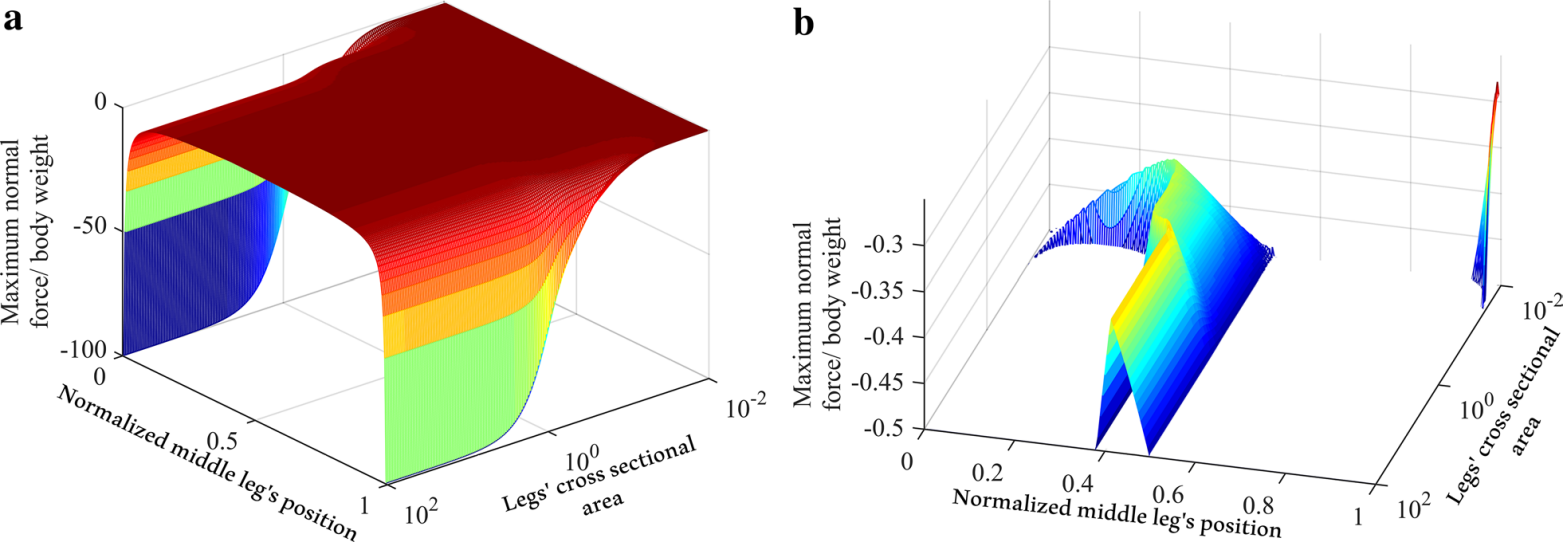
A range of values of legs’ cross-sectional area and middle leg’s positions, **a** maximum adhesion force requirement, and **b** the maximum adhesion force within −0.5 and −0.1 of the maximum normal force/body weight

The range of the cross-sectional area in Fig. 10 is selected to be from $0.0001$ to $3.16 \times 10^{4}$. Simulations performed considering the values of the cross-sectional area outside this range showed that variation of the cross-sectional area had little effect (variation smaller than 0.01 %) on the force distribution. The three sub-figures in Fig. 10 are combined to show the minimum normal forces among the front, middle and hind legs in Fig. 11, which represents the maximum adhesion required to keep the robot attached to the wall.

The best position for the middle leg, in the range between 0 and 0.99, is located between 0.3 and 0.42 for the range of legs’ cross-sectional area from 0.045 to 100, while the best range for smaller cross-sectional area, less than 0.045, jumps to be at 0.99, see Fig. 11b. For any cross-sectional area, the best position of the middle leg is when it overlaps the front leg, i.e., the middle leg has a position equal to one for any cross-sectional area value.

In summary, the optimal configuration when the body is parallel and the legs are perpendicular to the vertical surface is when the structure has a minimum legs’ cross-sectional area of $0.0001$ and a middle leg’s position of 0.99. Changing the body’s cross-sectional area and fixing the legs’ cross-sectional area have an opposite adhesion force requirement behavior to that shown in Fig. 10; the lowest point of the graph is when the body cross-sectional area is at minimum, which equals $10^{ - 4}$, and the maximum point is when the radius at maximum, which equals $3.16 \times 10^{4}$.

### Effect of middle leg position, height and legs’ cross-sectional area

**Fig. 12:**
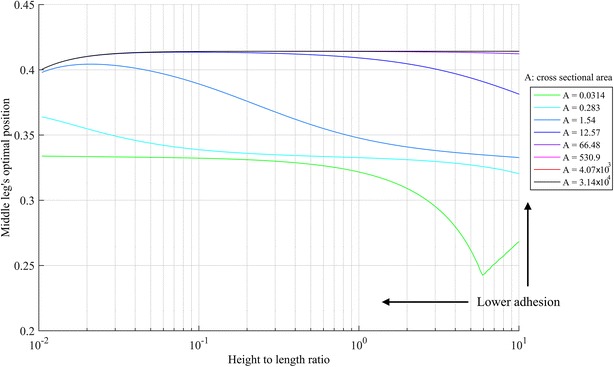
The optimal position of the middle leg for different heights to length ratios and different cross-sectional area of the structure. Lower height to length ratio results in lower adhesion requirement, while higher cross-sectional area also results in lower adhesion requirement

Previous results can be generalized for robots with different height to length ratios. In fact, an optimization is carried out to find the optimal middle leg position for different legs’ cross-sectional areas at different height to body length ratios, and the results are shown in Fig. 12. Similar to “Optimal middle leg position and height to length ratio” section, the optimizer is configured to search for the optimal middle leg’s position within the range of 0–0.95 to prevent the optimizer from converging to the undesired global optimum at 1.

The best middle leg’s position for a range of height to length ratios, chosen arbitrarily between 0.01 and 10, and different cross-sectional area between $0.0001$ and $3.16 \times 10^{4}$ is bounded between 0.24 and 0.41. Figure 12 allows the designer to identify the optimal middle leg’s position for different legs’ cross-sectional areas at different height to length ratios.

In Fig. 12, the best configurations are the ones with the lowest height to length ratio and it is found that the best configuration at a specific height is the one with the highest legs cross-sectional area value. In fact, the improvement in the adhesion for the entire cross-sectional areas investigated range, and the height to length ratios is calculated to be between 22 and 71 %. For example, at the specific height to length ratio of 1, arbitrarily chosen, the best adhesion requirement at the considered cross-sectional areas sorted from best to worse is: [3.14 × 10^4^ 4.07 × 10^3^ 530.9 66.48 12.57 1.54 0.283 0.0314], where the first four cross-sectional areas have almost the same adhesion requirement value and the improvement in the adhesion force requirement between the cross-sectional area of $3.14 \times 10^{4}$ and $0.0314$ is calculated to be 29 %.

In part 2 of the paper, the posture of living ants loitering on a vertical surface is used to confirm the validity of the assumptions used in this paper. The authors simplified the ants’ structure in the same manner as in this part 1. The model proposed in this part 1 paper was used to predict the configuration of the ants’ posture and the effect of both their middle leg’s position and body’s cross-sectional area.

## Conclusion

In this work, the effect of different geometrical parameters on the structure of a legged robot is investigated and analyzed using the stiffness method. To improve the efficiency of vertical climbing, the height to length ratio of the robot should be kept as low as possible, the cross-sectional area of the legs should be as big as possible, and the middle leg should be positioned between 0.24 and 0.41. The presented parametric study is utilized as an outline to assist designing the structure of climbing robots when loitering on a vertical surface. The presented results are applicable to any robot size as they are unit-less. Equation (6), which is at the foundation of the developed code, can potentially be implemented in a microcontroller to optimize in real time the posture of the robot. Results presented in this work yield the following guidelines to design climbing robots: (1) height to length ratio should be as small as possible; (2) the cross-sectional area of the structure should be as big as possible; and (3) the position of the middle leg can be selected from Fig. 12, which provides the optimal middle leg’s position as a function of the height to length ratio and the cross-sectional area of the structure.

## Appendix

In this Appendix we show that the use of different materials does not change the results of the geometrical optimization that is performed. In fact, the stiffness matrix for one beam is given by:

A1 $$\varvec{F} = \left[ {\begin{array}{*{20}c} {\frac{AE}{L}} & 0 & 0 & { - \frac{AE}{L}} & 0 & 0 \\ 0 & {\frac{12EI}{{L^{3} }}} & {\frac{6EI}{{L^{2} }}} & 0 & { - \frac{12EI}{{L^{3} }}} & {\frac{6EI}{{L^{2} }}} \\ 0 & {\frac{6EI}{{L^{2} }}} & {\frac{4EI}{L}} & 0 & { - \frac{6EI}{{L^{2} }}} & {\frac{2EI}{L}} \\ { - \frac{AE}{L}} & 0 & 0 & {\frac{AE}{L}} & 0 & 0 \\ 0 & { - \frac{12EI}{{L^{3} }}} & { - \frac{6EI}{{L^{2} }}} & 0 & {\frac{12EI}{{L^{3} }}} & { - \frac{6EI}{{L^{2} }}} \\ 0 & {\frac{6EI}{{L^{2} }}} & {\frac{2EI}{L}} & 0 & { - \frac{6EI}{{L^{2} }}} & {\frac{4EI}{L}} \\ \end{array} } \right] \cdot \varvec{D}$$

where $E$ is the elasticity coefficient, $A$ is the cross-sectional area of the beam, $L$ is the length of the beam and $I$ is the inertia of the beam. Assuming all of the cross sections of the beams is a circle; the second moment of inertia for a circular cross section is:

A2 $$I = \frac{{\pi r^{4} }}{4} = \frac{{Ar^{2} }}{4}$$

Factoring out $E$, $A$ and $L$ can be written as:

A3 $$\varvec{F} = \frac{{\varvec{A} \cdot \varvec{E}}}{\varvec{L}}\left[ {\begin{array}{*{20}c} 1 & 0 & 0 & { - 1} & 0 & 0 \\ 0 & {\frac{{3\varvec{r}^{2} }}{{\varvec{L}^{2} }}} & {\frac{{3\varvec{r}^{2} }}{{2\varvec{L}}}} & 0 & { - \frac{{3\varvec{r}^{2} }}{{\varvec{L}^{2} }}} & {\frac{{3\varvec{r}^{2} }}{{2\varvec{L}}}} \\ 0 & {\frac{{3\varvec{r}^{2} }}{{2\varvec{L}}}} & {\varvec{r}^{2} } & 0 & { - \frac{{3\varvec{r}^{2} }}{{2\varvec{L}}}} & {\frac{{\varvec{r}^{2} }}{2}} \\ { - 1} & 0 & 0 & 1 & 0 & 0 \\ 0 & { - \frac{{3\varvec{r}^{2} }}{{\varvec{L}^{2} }}} & { - \frac{{3\varvec{r}^{2} }}{{2\varvec{L}}}} & 0 & {\frac{{3\varvec{r}^{2} }}{{\varvec{L}^{2} }}} & { - \frac{{3\varvec{r}^{2} }}{{2\varvec{L}}}} \\ 0 & {\frac{{3\varvec{r}^{2} }}{{2\varvec{L}}}} & {\frac{{\varvec{r}^{2} }}{2}} & 0 & { - \frac{{3\varvec{r}^{2} }}{{2\varvec{L}}}} & {\varvec{r}^{2} } \\ \end{array} } \right] \cdot \varvec{D}$$

A4 $$\varvec{F} = \left[ {\frac{{\varvec{A} \cdot \varvec{E}}}{\varvec{L}} \cdot \varvec{K}_{{\mathbf{E}}} } \right] \cdot \varvec{D}$$

$\varvec{K}_{{\mathbf{E}}}$ is the stiffness matrix multiplied by $\frac{L}{{\varvec{A} \cdot \varvec{E}}}$. In the same way, $\frac{{\varvec{A} \cdot \varvec{E}}}{L}$ can be taken as a common factor from all of the elements of the stiffness matrix in Eq. (6), which can be rewritten as:

A5 $$\varvec{F}_{{\mathbf{u}}} = \left[ {\frac{{\varvec{A} \cdot \varvec{E}}}{\varvec{L}} \cdot \varvec{K}_{{{\mathbf{E}} 2 1}} } \right] \cdot \left[ {\frac{{\varvec{A} \cdot \varvec{E}}}{\varvec{L}} \cdot \varvec{K}_{{{\mathbf{E}}11}} } \right]^{ - 1} \cdot \varvec{F}_{{\mathbf{k}}}$$

A6 $$\varvec{F}_{{\mathbf{u}}} = \frac{{\varvec{A} \cdot \varvec{E}}}{\varvec{L}} \cdot \varvec{K}_{{{\mathbf{E}}21}} \cdot \frac{\varvec{L}}{{\varvec{A} \cdot \varvec{E}}} \cdot \varvec{K}_{{{\mathbf{E}}11}}^{ - 1} \cdot \varvec{F}_{{\mathbf{k}}}$$

the above equation can be written as:

A7 $$\varvec{F}_{{\mathbf{u}}} = \varvec{K}_{{{\mathbf{E}}21}} \cdot \varvec{K}_{{{\mathbf{E}}11}}^{ - 1} \cdot \varvec{F}_{{\mathbf{k}}}$$

It can be seen that equation (A7) is independent of the elasticity coefficient $E$. In other words, the elasticity of the beams relative to each other is what causes the change in the force distribution.
